# A high vascular count and overexpression of vascular endothelial growth factor are associated with unfavourable prognosis in operated small cell lung carcinoma

**DOI:** 10.1038/sj.bjc.6600130

**Published:** 2002-02-12

**Authors:** G Fontanini, P Faviana, M Lucchi, L Boldrini, A Mussi, T Camacci, M A Mariani, C A Angeletti, F Basolo, R Pingitore

**Affiliations:** Department of Oncology, Transplants and Advanced Technologies in Medicine, University of Pisa, via Roma 57, 56126 Pisa, Italy; Department of Cardio-Thoracic Surgery, University of Pisa, via Roma 57, 56126 Pisa, Italy; Department of Surgery, University of Pisa, via Roma 57, 56126 Pisa, Italy

**Keywords:** angiogenesis, VEGF, small cell lung cancer

## Abstract

It has been widely demonstrated that neo-angiogenesis and its mediators (i.e. vascular endothelial growth factor), represent useful indicators of poor prognosis in non small cell lung carcinoma. In order to verify whether neovascularization and vascular endothelial growth factor may be considered useful markers of clinical outcome also in the small cell lung cancer subgroup, we retrospectively investigated a series of 75 patients with small cell lung carcinoma treated by surgery between 1980 and 1990. Immunohistochemically-detected microvessels and vascular endothelial growth factor expressing cells were significantly associated with poor prognosis, as well as with nodal status and pathological stage. In fact, patients whose tumours had vascular count and vascular endothelial growth factor expression higher than median value of the entire series (59 vessels per 0.74 mm^2^ and 50% of positive cells, respectively), showed a shorter overall and disease-free survival (*P*=0.001, *P*=0.001; *P*=0.008, *P*=0.03). Moreover, the presence of hilar and/or mediastinal nodal metastasis and advanced stage significantly affected overall and disease-free interval (*P*=0.00009, *P*=0.00001; *P*=0.0001, *P*=0.00001). At multivariate analysis, only vascular endothelial growth factor expression retained its influence on overall survival (*P*=0.001), suggesting that angiogenic phenomenon may have an important role in the clinical behaviour of this lung cancer subgroup.

*British Journal of Cancer* (2002) **86**, 558–563. DOI: 10.1038/sj/bjc/6600130 www.bjcancer.com

© 2002 Cancer Research UK

## 

Small cell lung cancer (SCLC) represents one of the most aggressive subgroups of lung cancer, and, despite its initial sensitivity to chemotherapy, most patients with this type of cancer die of metastatic disease in less than 2 years from diagnosis (Brigham *et al*, 1978). In the last two decades a large number of clinical studies have indicated that the angiogenic pattern may be a useful indicator of clinical outcome of tumours, including lung cancer (Weidner, 2000). In fact, much data has shown that the number of microvessels in the tumour area, as an index of neo-angiogenesis, correlates to an increased risk of metastatic disease and a worse overall survival (OS) (Macchiarini *et al*, 1992; Apolinario *et al*, 1997; Fontanini *et al*, 1997a). A number of factors are known to stimulate new vessel formation, of which vascular endothelial growth factor (VEGF) is the most commonly expressed in human tumours (Ferrara, 1995). VEGF has been shown to increase tumour growth and angiogenesis *in vivo* in a nude mice model (Ferrara *et al*, 1993; Zhang *et al*, 1995). Similarly, anti-VEGF antibodies have the ability to inhibit the growth and dissemination of several tumour cell lines in nude mice (Kim *et al*, 1993).

Recent studies in NSCLC have shown that both vascular count and VEGF expression may influence progression and clinical outcome of the tumours (Fontanini *et al*, 1997a,b, 1999). Moreover, some interesting data indicate a putative genetic control of neo-angiogenesis by genes involved in the proliferative/apoptotic balance (i.e. p53 and bcl-2) (Bouck, 1996; Fontanini *et al*, 1997c). There is also evidence that inhibition of angiogenesis limits tumour growth by promoting apoptosis (Holmgreen *et al*, 1995).

SCLC is rarely treated by surgery, and, as a consequence, not enough data are available for an exhaustive biological characterization of this type of cancer. Recent analysis has correlated the clinical outcome of SCLC series to vascular count and VEGF serum levels, although discrepancies have been observed in the results obtained in these studies (Salven *et al*, 1998; Eerola *et al*, 2000).

In order to investigate the role of neo-angiogenesis in the outcome of SCLC, we analyzed vascular count, VEGF expression and p53 pattern in a series of resected SCLC, with interesting results from a prognostic point of view. The association we found in our study between VEGF protein expression and outcome is very suggestive of a putative role of this factor in the development and progression of the SCLC subgroup, although no confirmatory data are now available. Despite progression in surgery and chemotherapy, prognosis of SCLC remains unfortunately extremely bad, and new therapeutic approaches would be hoped for. In this sense, the finding of new biologic factors related to progression and poor outcome may represent a useful target of innovative therapeutic tools, such as anti-angiogenic therapies. In this respect, SCLC may be an ideal field in which to test new anti-angiogenic therapies in association with conventional chemotherapies.

## MATERIALS AND METHODS

### Patients

Seventy-five SCLC patients who had undergone curative surgical resection at the Service of Thoracic Surgery, University of Pisa, between 1980 and 1990, were analyzed. There were 72 males and three females (mean age 61.7 years, median 62 years, range 34–75 years). All the patients underwent a complete preoperative staging. This included: a detailed history and a physical examination, the evaluation of the Performance Status according to Karnofsky, a complete blood count and biochemical profile, cardiac and pulmonary function tests, chest X-ray, bronchoscopy, computed tomography of chest, the upper abdomen and brain, abdominal ultrasonography, bone scan, Gallium-67 scan, bilateral bone marrow biopsy and aspiration. Preoperative mediastinoscopy was not routinely performed where there was an absence of Gallium up-take on the mediastinum and no bulky mediastinal lymph-node involvement was evident. All the patients received surgery as their first line therapy, followed by adjuvant chemotherapy and, in the cases of hilar or mediastinal involvement, also by radiotherapy. According to Tumour-status, there were 16 T1, 48 T2, and 11 T3; 41 patients did not show nodal metastasis at the moment of diagnosis, whereas 34 showed hilar and/or mediastinal metastatic involvement. Data on clinical behaviour were available in all of the 75 cases (median follow-up 109.6 months, range 25–222). Twenty-nine of the patients were alive at the moment of analysis, whereas 46 were dead. Tumours were classified in accordance with the World Health Organization (WHO, 1982) classification and within the guidelines of the American Joint Committee for Cancer Staging (AJCCS, 1992).

### Immunohistochemistry

#### Microvessel detection and counting

The method of microvessel detection and counting (MVC) has been described previously (Fontanini *et al*, 1997a). Briefly, intratumour microvessels were highlighted with anti-CD34 Mab (QB-END 10, Novocastra Laboratories, Newcastle, UK) diluted 1 : 100, after heating the sections in a microwave oven twice for 5 min at 700 W in citrate buffer (pH 7.6). Biotinylated anti-mouse IgG (Vector, Burlingame, CA, USA) was applied for immunoreaction, followed by detection using the ABC method. In all cases, MVC was determined independently by two pathologists. Each pathologist evaluated the slides without any knowledge of the counts made by the other pathologist and of the clinical outcome of the patients. No significant differences in MVC were found between the two observers. In fact, the counts from the two pathologists showed a very good correlation (*r*=0.98; two-sided *P*=0.0001). When conflicting data were obtained, we used mean values. A single microvessel was defined as any brown, immunostained endothelial cell that was separated from adjacent microvessels, tumour cells, and connective tissue elements. Each sample was examined by each pathologist, who identified the three most intense regions of neovascularization under low microscopic power (×10 objective lens and ×10 ocular lens). An ×250 field (×25 objective lens and ×10 ocular lens; 0.74 mm^2^ per field) in each of these three areas was then counted, and the average count of the three fields was recorded. Large vessels with thick, muscular walls were excluded from the counts. The presence of a lumen was not required to identify a microvessel. MVCs were categorized as a dichotomous variable (*low* *vs* *high* MVC). In the dichotomous categorization a count of 59 microvessels (the median value obtained in this series) was used as the cut-off point to distinguish a low MVC from a high MVC.

#### VEGF expression

In 53 out of 75 of the cases, according to the availability of the tumoural material, immunostaining for VEGF was performed using the ABC method in formalin-fixed, paraffin-embedded tissue samples. Sections were dewaxed in xylen, taken through ethanol, and then incubated with 0.3% hydrogen peroxidase in methanol for 10 min to block the endogenous peroxidase activity. After washing with phosphate-buffered saline (PBS) and incubation for 30 min with 10% normal goat serum, the sections were incubated overnight with anti-VEGF monoclonal antibodies (SantaCruz Biotechnology, Inc., Santa Cruz, CA, USA, dilution 1 : 50). Anti-VEGF is raised against a synthetic peptide corresponding to amino acid residues 1 to 191 of human VEGF; it recognizes the 165, 189 and 121 amino splicing variants of VEGF. After the primary antibodies, biotynilated anti-rabbit IgG (Vector Laboratories, Burlingame, CA, USA) were applied and followed by detection using the ABC method (Vector Laboratories, Burlingame, CA, USA). Light counterstaining was performed with haematoxylin. Normal rabbit immunoglobulin G substituted the primary antibody as negative controls. VEGF expression was evaluated as percentage of positive cells in a total of at least 1000 tumour cells. Tumour sections with no VEGF immunoreactive cells were considered as negative. The median values of the series (50% of positive) was used as cut-off values to distinguish low from high VEGF expressing tumours.

#### P53 expression

As well as VEGF expression, the p53 protein immunostaining was assessed in formalin-fixed and paraffin-embedded tissue samples using immunohistochemistry. NCL-DO7 anti-p53 monoclonal antibody (Novocastra Laboratories, New Castle, UK) was used to detect the p53-altered protein (1 : 250 of dilution). The avidin-biotin peroxidase method was used by developing immunoreaction with Diaminobenzidine. Simultaneous staining of a known p53 positive case was employed as positive control for p53. Incubation of parallel slides omitting the first antibody was performed as negative control. The count of p53 immunoreactive cells was made by scoring a minimum of five high – power fields (HPFs) (40× objective lens) and counting the number of immunoreactive cells out of the total of epithelial cells analyzed in each field. The 5% of positive cells was used as cut-off value to distinguish negative from positive tumours.

### Statistical analysis

All statistical analyses were carried out using the Statistica and SPSS softwares. Univariate analysis was performed by modelling Kaplan–Meier survival curves. The Log-rank test was used to evaluate the statistical significance of differences in survival distributions among prognostic groups. Multivariate analysis was carried out by use of the Cox proportional-hazard model. The Cox model was first used to select from among variables that significantly affected survival in univariate analysis, and then from among those variables whose prognostic role was independent.

## RESULTS

### Clinico-pathological parameters and OS

Among the clinico-pathological parameters, metastatic nodal-involvement (*P*=0.00009), and advanced stage (*P*=0.0001) were significantly associated with a worse overall survival. No association was found between bad prognosis and tumour-status ( Table 1

**Table 1:**
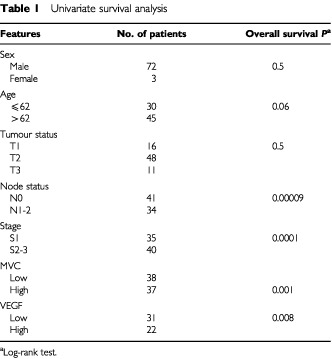
Univariate survival analysis

). A similar statistically-significant association was observed between these characteristics and disease-free survival (data not shown). Figure 1

**Figure 1:**
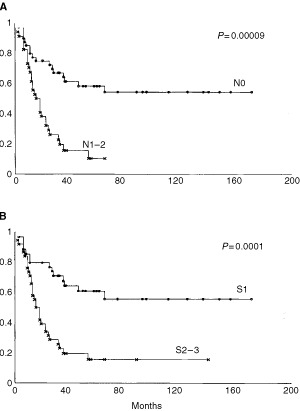
Kaplan–Meier OS estimates in relation to Nodal Status (**A**) and Stage (**B**).

shows Kaplan–Meier survival plots generated on the basis of nodal status (Figure 1A), and stage (Figure 1B).

### Vascular count and outcome in SCLC

**Figure 2:**
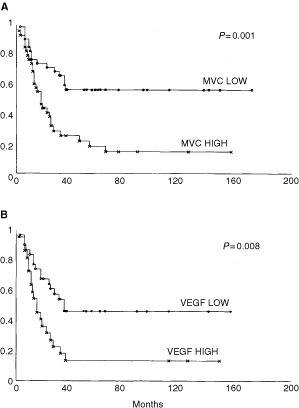
Kaplan–Meier OS estimates in relation to MVC (**A**) and VEGF expression (**B**). A MVC of 59 microvessels was the median cut-off in the series of 75 SCLC patients for considering low or high tumour angiogenesis.

The number of microvessels, evaluated as mean in the three areas of each tumour with most intense vascularization, showed a mean of 63.4 vessels and a median value of 59 vessels (range 14–170 vessels). MVC, analyzed as a dichotomous variable (using the median value of 59 microvessels as the cut-off point), was a highly significant predictor of both overall survival (Table 1; *P*=0.001) and of disease-free survival (data not shown). Figure 2

shows Kaplan–Meier survival plots generated on the basis of low and high vascular count (Figure 2A).

### VEGF and p53 expression and outcome in SCLC

VEGF protein expression, evaluated as low and high on the basis of median value of the series (mean 40.1, range 0–90%), significantly affected overall (*P*=0.0008) and disease-free survival. Figure 2 shows Kaplan–Meier survival plots generated on the basis of low and high VEGF expression (Figure 2B). No statistical association was found between p53 alterations and overall survival. In fact, tumours expressing p53 protein failed to influence the clinical behaviour of the disease (*P*=0.1). Furthermore, no association was found between p53 alterations, vascular count and VEGF expression (*P*=0.6; *P*=0.8) (data not shown).

### Vascular count, VEGF expression and clinico-pathological parameters

When we analyzed the relationship between vascular count and VEGF expression, clinico-pathological parameters such as sex and age of patients, tumour-status, nodal-status and stage, we were unable to find any statistical association among these variables (data not shown). Moreover, no significant differences were found between MVC and VEGF expression and site of tumours.

### Multivariate analysis

A multivariate analysis was performed to evaluate the independent prognostic role of MVC after adjusting for other significant covariates. All variables that significantly affected survival in univariate analysis were introduced into a Cox proportional-hazard model ( Table 2

**Table 2:**
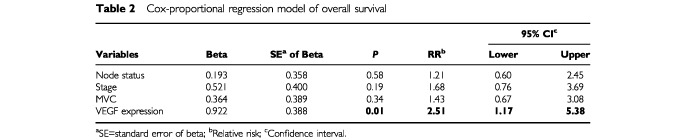
Cox-proportional regression model of overall survival

). At the end of the stepwise process, only VEGF expression maintained its independent prognostic influence on overall survival (*P*=0.01). In this analysis, vascular count failed to show any independent influence on overall survival (OS).

## DISCUSSION

The importance of tumour angiogenesis in tumour development and progression has been revealed in recent years. Several studies (revised in Weidner and Folkman, 1996) have suggested that MVC (as a measure of tumour angiogenesis) is a significant predictor of increased risk of metastatic disease and worse OS in several types of human cancer, including lung cancer. However, relatively few, and no conclusive reports have been published on the prognostic significance of MVC in lung cancers (Macchiarini *et al*, 1992; Harpole *et al*, 1996; Giatromanolaki *et al*, 1996), although in the NSCLC subgroup some important results have been obtained (Fontanini *et al*, 1997a). Neo-angiogenesis is under the control of several growth factors and cytokines (Bicknell and Harris, 1991), and the imbalance between stimulating and inhibiting factors is responsible for activating the angiogenic potential of the tumours. VEGF plays a crucial role in the control of angiogenesis both in physiological and pathological situations (Shibuya, 1995), including tumour development and progression. It is mitogenic and angiogenic for endothelial cells, and it can also increase vascular permeability.

A number of tumour cells in humans have been seen to express higher levels of VEGF mRNA compared to normal tissue (Brown *et al*, 1993; Plate *et al*, 1993; Abu-Jawdeh *et al*, 1996; Yoshiji *et al*, 1996), suggesting an association of VEGF with a malignant phenotype; furthermore, an elevated VEGF expression has also been correlated to a worse prognosis in cancers such as gastric (Maeda *et al*, 1996; Takahashi *et al*, 1996), colonic (Takahashi *et al*, 1995) and lung carcinomas (Mattern *et al*, 1995; Ohta *et al*, 1996; Fontanini *et al*, 1997b, 1999).

Small cell lung carcinomas represent a subgroup of lung cancer which substantially differ from the non-small cell subtype. In fact, the behaviour of this group of cancers is extremely bad, despite its initial sensitivity to chemotherapy. An exhaustive biological characterization of SCLC is difficult because of the different therapeutic approach usually employed in this type of tumour: SCLC is rarely treated by surgery, and most centres have few specimens on which to perform a biological characterization. Previously, we published our surgical experience and our policy in the clinical selection of patients for a multimodality treatment including surgery in SCLC-limited disease (Lucchi *et al*, 1997). Surgery can be applied as part of a combined treatment in patients with limited SCLC, selected on the basis of TNM staging. Moreover, despite conventional imaging and invasive procedures (mediastinoscopy etc.), the clinical prediction of lymphnodal involvement is unsatisfactory, with about 40% of more advanced disease at operation. This is the major obstacle in the correct planning of the multimodality treatment of limited-disease SCLC, and the reason for it's being inappropriate to compare the results of surgical treatments with the chemo-radiotherapy and neoadjuvant treatments. In order to verify the role of angiogenic phenomenon also in SCLC, as we had previously performed in a large series of non-small cell lung tumours (Fontanini *et al*, 1997a,b,c), a series of 75 SCLC was investigated, with particular reference to vascular count, VEGF and p53 expression. In our series we observed that nodal metastasis and tumour stage were important predictors of poor prognosis, as already reported in previous studies (Shepherd *et al*, 1993).

As regards vascular count, we observed that a higher number of microvessels (higher than median value of the series) in the most intense area of neovascularization significantly affected overall and disease-free survival. However, although exciting, these findings are in contrast with results recently presented by Eerola *et al* (2000). In a series of 56 SCLC they failed, in fact, to demonstrate any significant association between outcome and microvessel density. This discrepancy may be mostly due to a different methodology used for detecting microvessels in tumour areas. Eerola *et al* (2000) in fact, immunostained their samples by an anti-FVIII polyclonal antibody, that, although very specific, may be a bit less sensitive than the anti-CD34 monoclonal antibody we used, and thus causing a misinterpretation of the number of microvessels. A median value of nine microvessels per HPF was used by the authors for distinguishing a low from a high vascular density, dramatically far from the median value we found using CD34 monoclonal antibody in our series of cancers. However, also from our observations we believe that vascular density in SCLC has a different prognostic impact in comparison with vascular density in NSCLC. Indeed, in multivariate analysis, vascular count did not retain its influence on OS also in our study. However, an interesting aspect of our paper is related to the expression of VEGF, which represents one of the most important factors of vascular development and growth. Previous results of ours demonstrated that both protein and mRNA VEGF expression were strictly associated with prognosis in the NSCLC subgroup (Fontanini *et al*, 1997b, 1999). In our current study we observed that a high VEGF expression was significantly associated with poor prognosis and, most important, that in multivariate analysis VEGF expression retained its prognostic significance on OS. Interestingly, recent data from Salven *et al* (1998) in SCLC showed that pre-treatment serum levels of VEGF were associated with poor response to treatment and unfavourable survival in patients treated with combination chemotherapy with or without interferon. VEGF is an important mediator of tumour-induced angiogenesis and represents a potential target for innovative anticancer therapies. Starting from this concept, and with it's having recently been demonstrated in experimental models that anti-VEGF treatment augments tumour radiation response and the efficacy of cancer immunotherapy (Gabrilovich *et al*, 1999; Lee *et al*, 2000), the evaluation of VEGF and its receptors may represent a useful aim in drawing up a correct therapeutic profile of SCLC patients. In several animal models, neutralizing anti-VEGF antibodies have shown encouraging inhibitory effects on solid tumour growth, ascites formation and metastatic dissemination. Several of these anti-VEGF therapies are currently being tested in clinical trials in cancer patients. For these specific reasons a careful assessment of the anti-angiogenic profile of a tumour seems very interesting, particularly in those cases in which pharmacological approaches represent an important modality of treatment.

As regards p53 protein alterations, we did not observe in 51 of the cases analyzed any association between these alterations and vascular count and/or VEGF expression, although a high percentage of p53 mutations have been reported to be very frequent also in SCLC (Takahashi *et al*, 1991; Levin *et al*, 1994). Probably in this type of cancer, the genetic regulation of neo-angiogenesis is different from what we observed in the NSCLC subgroup, suggesting the possibility of more complex mechanisms and multiple interactions of several mediators.

Taken together, these data suggest the essentiality of further analyzing the complex phenomenon of neo-angiogenesis also in SCLC, in order to determine if in this model the vascular network and/or its regulators may modify in some way the behaviour of this type of cancer, and whether, in SCLC, neo-angiogenesis may represent an ideal field in which to test new anti-angiogenic drugs in association with chemotherapy.
